# LncRNAs as the Regulators of Brain Function and Therapeutic Targets for Alzheimer’s Disease

**DOI:** 10.14336/AD.2021.1119

**Published:** 2022-06-01

**Authors:** Yuqing Liu, Xin Chen, Yutong Che, Hongli Li, Zheyu Zhang, Weijun Peng, Jingjing Yang

**Affiliations:** ^1^Department of Integrated Traditional Chinese & Western Medicine, The Second Xiangya Hospital, Central South University, Changsha, Hunan, China.; ^2^Xiangya School of Medicine, Central South University, Changsha, Hunan, China.; ^3^Inter-disciplinary Research Center of Language Intelligence and Cultural Heritages, Hunan University, Changsha, Hunan, China.; ^4^Teaching and Research Section of Clinical Nursing, Xiangya Hospital, Central South University, Changsha, China.; ^5^Xiangya Nursing School, Central South University, Changsha, China.

**Keywords:** Alzheimer’s disease;, lncRNAs, targeted drugs, exosome

## Abstract

Alzheimer’s disease (AD) is the most common type of dementia and a serious threat to the health and safety of the elderly population. It has become an emerging public health problem and a major economic and social burden. However, there is currently no effective treatment for AD. Although the mechanism of AD pathogenesis has been investigated substantially, the full range of molecular factors that contribute to its development remain largely unclear. In recent years, accumulating evidence has revealed that long non-coding RNAs (lncRNAs), a type of non-coding RNA longer than 200 nucleotides, play important roles in multiple biological processes involved in AD pathogenesis. With the further exploration of genomics, the role of lncRNA in the pathogenesis of AD has been phenotypically or mechanistically studied. Herein, we systematically review the current knowledge about lncRNAs implicated in AD and elaborate on their main regulatory pathways, which may contribute to the discovery of novel therapeutic targets and drugs for AD.

Alzheimer’s disease (AD), also known as senile dementia, is a neurodegenerative disease with insidious onset and chronic progressive course. Symptoms such as progressive memory impairment, cognitive dysfunction, personality changes, language dysfunction, and basic behavioral dysfunction greatly interfere with patients’ social, life, and work functions [1]. The prevalence rate of AD in China is about 3.1%. Currently, there are about 50 million people with AD worldwide, and about one-third of the cases occur in China. The onset of the disease is slow and insidious, and patients and their families often cannot tell when the onset occurs. It is more common in patients aged >70 years (male average age: 73 years, female average age: 75 years). The female-to-male ratio is 3:1. The pathogeny and mechanism of AD have not yet been clarified. The pathological process is characterized by senile plaques formed by deposition of amyloid-β (Aβ) protein, neurofibrillary tangles formed by hyper-phosphorylation of Tau protein, and neuronal loss accompanied by glial cell proliferation [2, 3]. In the past two decades, an increasing number of scientists have shown great interest in the molecular basis of AD, but the actual pathogenesis and etiology are not well understood, as it is believed that the disease is a neurodegenerative condition guided by inflammation in the brain[4]. Although effective medications can improve symptoms and quality of life, there are currently no drugs that can cure or prevent the development of cognitive impairment. Verubecestat, for example, inhibits BACE1 (β-amyloid precursor protein lyase 1) and reduces Aβ protein in the cerebrospinal fluid of healthy people and AD by about 60%; however, verubecestat does not improve cognitive function at any dose [5]. Therefore, it is imperative to determine the etiological factors and mechanism of AD to develop a novel therapy for AD.

Long non-coding RNA (lncRNA) is a regulatory RNA transcript that is >200 nucleotides in length [6]. It has similar features to mRNA, but almost no open reading frame and therefore no translation function. LncRNAs were initially thought to be transcriptional disruptors, but are now reported to play important roles in the regulation of gene expression, including pre-transcriptional chromatin modification, transcriptional activation, transcriptional arrest, and post-transcriptional regulation [7]. With the maturity of high-throughput sequencing and lncRNAs characterization-based experimental technologies, the role of some lncRNAs in regulatory pathways in the development of various biological diseases has also been verified.

In recent years, the probable pathogenic effects of lncRNAs in AD have been explained owing to the increasing number of identified lncRNAs, which have been reported to regulate the pathological change in AD. Here, we discuss the roles of lncRNAs and their effects in AD, given that dysfunctions in lncRNA are likely a pathogenic mechanism associated with neurodegenerative processes [8, 9]. In addition, for a comprehensive and handy evaluation of the relevant lncRNAs, the existing major databases are listed in Table 1. Furthermore, the accuracy of diagnosis and potential treatment is discussed to help overcome the major challenges in neurobiology, although an effective treatment for AD is currently non-existent [10].

**Table 1:** LncRNAs research resources.

Database name	Website	Key Features
HGNC	https://www.genenames.org/	Name the lncRNAs
GENCODE	http://www.gencodegenes.org/	15779 human lncRNA genes were annotated with high precision
LNCipedia	http://www.lncipedia.org/	Gene secondary structure information, coding potential and miRNA binding site prediction
NONCODE	http://www.noncode.org/	Collect 17 species of lncRNA gene annotation information
lncRNAtor	http://lncRNAtor.ewha.ac.kr/index.htm	Integration derives from the lncRNA information of ENSEMBL, HGNC, MGI and lncRNAdb, providing co-expression analysis of mRNA and lncRNA
LncRNASNP2	http://bioinfo.life.hust.edu.cn/lncRNASNP/	Comprehensive database of single nucleotide polymorphisms (SNPs) in lncRNAs
DIANA-LncBase	http://carolina.imis.athena-innovation.gr/	Functional interactions of mirNA-lncrNA and conserved analysis
lncRNAdb	http://www.lncRNAdb.org/	Comprehensive annotation of lncRNA sequence structure information, genomic background, expression, subcellular localization, conserved and functional prediction
LncDisease	http://www.cuilab.cn/lncRNAdisease/	LncRNA-disease association data to predict lncRNA-related diseases
starbase	http://starbase.sysu.edu.cn/starbase2/	Construct ceRNA network
LncRscan-SVM	https://sourceforge.net/projects/lncrscansvm/	Characterization of source genes, potential codon sequences and conservativeness
LncRNA-MFDL	https://omictools.com/lncRNA-mfdl-tool	Identify the lncRNA
LncRNA-ID	https://omictools.com/lncRNA-id-tool	Identification of lncRNA
LncBook	http://bigd.big.ac.cn/lncbook/lncRNAs	The most abundant human lncRNA database

## 1. Overview of lncRNAs

### 1.1 Basic structure

LncRNAs are molecules that range from 200 nucleotides to hundreds of kilobases[6] and can be divided into the following five types: antisense lncRNA, intronic transcript, large intergenic noncoding RNA (lincRNA), promoter-associated lncRNA, and UTR-associated lncRNA[7]. LncRNA functions are based on its primary structure (nucleotide sequence), secondary structure, tertiary structure, and higher structure. Many lncRNAs can trans bind with chromatin-modifying enzymes to form a double stem loop structure [11]. Cloverleaf secondary structures similar to tRNAs were found in many different regions of various lncRNAs.

**Figure 1.**
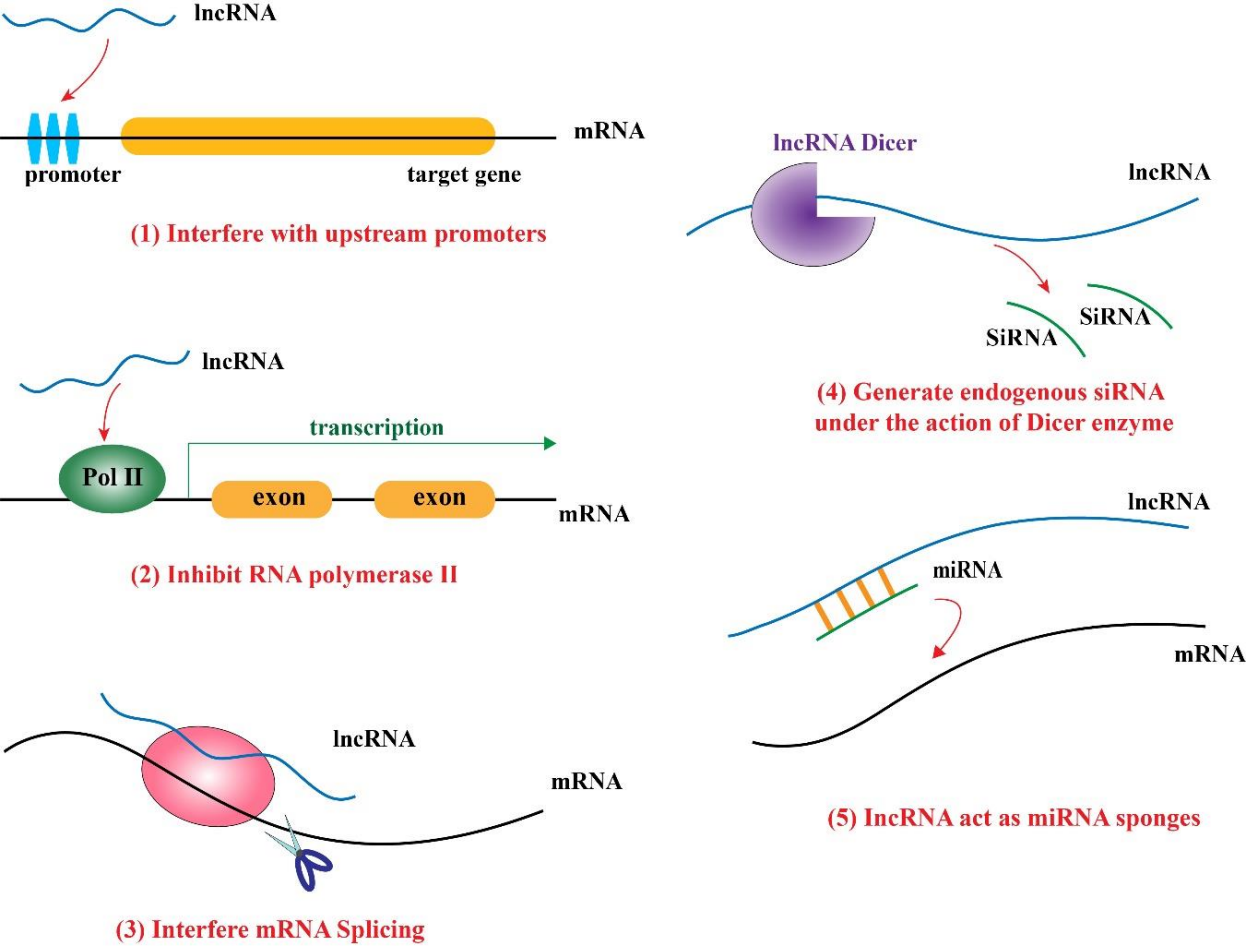
Mechanism of lncRNAs.

### 1.2 Biology

LncRNAs are usually long and have an mRNA-like structure. After splicing, they have a polyA tail and promoter structure, and have dynamic expression and different splicing methods during differentiation. However, they generally lack protein-coding functions, and unlike miRNA or piRNA, lncRNAs are highly heterogeneous, with different sizes, interaction partners, and modes of action. LncRNA are mainly located in the nucleus, but can also travel into the cytoplasm. Although the gene structure of lncRNA is similar to protein-coding genes [12], lncRNAs tend to have only one intron, and the co-transcriptional splicing efficiency is low. LncRNA can participate in post-translational regulation, form scaffolds, assemble functional ribonucleoprotein complex, and affect protein stability [13]. Most lncRNAs have obvious spatio-temporal expression specificity in the process of tissue differentiation and development. For example, research on lncRNAs in rats found that lncRNAs have various expression patterns in different parts of the brain tissue [14]. They are distributed differently across various brain regions and are associated with cell type, synaptic plasticity, and memory. Thus, lncRNAs are likely to play a crucial role in neurodegenerative diseases including AD.

### 1.3 Mechanism of lncRNAs

Over the past decade, several studies have highlighted that lncRNAs participate in various physiological processes of nuclear genes such as DNA replication, transcription, and post-transcriptional regulation, as well as epigenetic functions. Furthermore, lncRNAs perform significant cytoplasmic functions such as promoting or inhibiting the stability of mRNA and newborn proteins, as well as regulating translation and miRNA activity [15, 16].

The major targets and mechanisms of lncRNA are as follows (Fig. 1):

(i) Transcription of the upstream promoter region of the protein-encoding gene interferes with the expression of downstream genes [17]; (ii) Inhibiting RNA polymerase II to affect downstream gene expression [18]; (iii) Form complementary double chains with the transcript of protein encoding gene, disrupt the splicing of mRNA, and form various splicing forms [19]; (iv) Generate endogenous siRNA with the assistance of Dicer enzyme; (v) Act as an RNA sponge for small RNAs such as miRNA. LncRNAs can also act as a mediator, playing the role of a molecular scaffold, molecular guide, molecular bait, and signal pathway regulator, in addition to maintaining telomere stability. For example, GUARDIN acts as a molecular sponge to adsorb Mir-23A and regulate the expression of telomere repeat sequence binding factor (TRF2) to maintain the structural integrity of the chromosome terminal [20].

### 1.4 Effects of lncRNAs on neurodevelopment

Early research on lncRNA expression in mouse embryonic stem cells (ESCs) indicated that the expression of lncRNA was highly specific and strictly regulated with the differentiation of specific brain cell types. LncRNA plays a crucial role in the self-renewal ability and pluripotent differentiation of ESCs. Nuclear-localized lncRNAs can regulate gene transcription by binding or targeting specific chromatin modifiers or as scaffold structures, while plastic-localized lncRNAs can interact with microRNA to regulate the pluripotency of ESCs. For example, lncRNA can be absorbed as a sponge structure or combined with some microRNAs to interfere with the function of microRNA to regulate protein translation, and then adjust the multifunction of ESCs [7, 21].

After brain development, lncRNA is still the main controller of gene expression regulation network. Studies in mice have shown that mouse neurons enriched with lncRNAs exert extensive control over physiological behavior in the brain [22]. Progress in cell isolation techniques and tissue imaging techniques has enabled us to better investigate the effects of lncRNA profiles on cell specificity. Improvements in sequencing technology have enabled the first attempt to map the microregions of the human brain with single-cell resolution [23, 24]. Johnson et al. [25] found that mRNA expression patterns of individual cells can be used to identify subtypes of neurons or glial cells. Next, we analyzed the lncRNA composition of the cells, which showed that the high abundance of most lncRNAs in at least one type of brain cell during brain development, including radial glial cells from different brain structures and various neurons, confirms that lncRNAs play a role in the formation and maintenance of cellular characteristics and phenotypes.

### 1.5 Extracellular transport of lncRNA

Studies have shown that the effect of lncRNAs on gene expression may not be limited to the cells that originally produced them [26, 27]. This association suggests that extracellular vesicles mediate information exchange and communication between cells via transferred lncRNAs [28], such as between neurons and glial cells. Some lncRNA species in extracellular vesicles appear to be related to sequence motifs recognized by RNA-binding proteins. These have been observed in blood cells, and it is unclear whether an analogous mechanism exists in neurons. This shows that lncRNAs have therapeutic potential on neurons [29].

### 1.6 LncRNA gene-knockout model

Although only a few lncRNAs have been researched in terms of their effects and mechanisms of action, transgenic organisms with inducible or constitutionally deficient enzymes related to lncRNA biosynthesis play a crucial role in understanding the basic role of lncRNAs in brain function. Previous studies have shown that the CRISPR/Cas9 system can efficiently knock out the promoter region of the lncRNA gene, and this knockout can be inherited by the next generation. In addition, targeted inactivation of the *MALAT1* gene in a transgenic mouse model has also been used to study the role of lncRNA MALAT1 in breast cancer[30]. These findings provide an effective gene editing tool to study the function of lncRNA.

## 2. LncRNAs after brain injury in AD

### 2.1 Regulation of Aβ aggregation

Amyloid precursor protein (APP) is a type of single transmembrane protein that widely exists in tissues and cells throughout the body. APP produces the toxic Aβ protein after cleavage by protease. In the nervous system, it is primarily involved in synaptic formation and neuroplasticity. Many scholars believe that the senile plaques formed by Aβ deposition after APP lysis are an important cause of AD pathogenesis. It has been reported [31] that some lncRNAs can directly reduce APP biosynthesis as well as Aβ plaque formation.

#### 2.1.1 LncRNA BACE1-AS

BACE1- AS (Table 2) is the non-coding antisense RNA of BACE1 (Beta Amyloid Cleaving Enzyme1), an important aspartic protease that forms myelin in peripheral nerve cells. It is a transmembrane protein containing two active sites and can form dimers in the extracellular domain. It was found that deposition of Aβ in the brain increases the level of BACE1-AS; thereby increasing BACE1 mRNA and BACE1 in nerve tissue, which further increases Aβ formation [32-35] (Figure ).

Zeng et al.[36] found that BACE1-AS can act as competitive endogenous RNA, regulating the expression of BACE1 and improving the stability of BACE1 mRNA by binding its complementary miRNA fragment to BACE1 mRNA. At the same time, increased BACE1-AS level consumes miRNAs targeting BACE1 and BACE1-AS, resulting in the inhibition of BACE1 and excessive production of Aβ, which participates in the occurrence of AD. Bahn et al. [37] found that nuclear factor erythroid-derived 2-related factor 2 (NRF2) also participated in the development of AD through BACE1-AS. It inhibited the expression of BACE1 and BACE1-AS by binding to ARE in mouse promoters, decreasing the formation of BACE1, BACE1-AS transcripts, and the formation of Aβ, and specifically mediated cognitive impairment in animal models of AD. Currently, BACE1-AS has been verified to remain in a state of up-regulated expression in nerve tissue when a person has AD, and there are considerable differences in the distribution and concentration of BACE1-AS in plasma or plasma-derived exosomes between AD patients and healthy controls. In 2020, Wang et al. found that BACE1-AS was more abundant in the patient group than the control group. In their study, the receiver operating characteristic (ROC) curve analysis showed that BACE1-AS distinguished between pre-AD and healthy controls (75% sensitivity, 100% specificity) and between AD and healthy controls (68% sensitivity, 100% specificity). Plasma BACE1-AS level is expected to be an effective blood biomarker for the assessment of AD [38]. Some other studies have reported that BACE1-AS can be an important target to study the etiopathogenesis and therapies of AD [39-41].

**Table 2:** LncRNA dysregulation in Alzheimer's disease.

LncRNAs	Describe	Up and down	Biologic Function
BACE1-AS	Transcribe from the antisense protein- coding BACE 1 gene	Up	Increase BACE1 mRNA stability resulting additional A42 generation through a post- transcriptional feed-forward mechanism
BC200	Homologous with rodent BC1 lncRNA,the earliest specific example showedlncRNAs conservation	Soma: UpDendritic:Down	Modulate local proteins in postsynaptic dendritic microdomains to maintenance of long-term synaptic plasticity
NEAT1	Nuclear enriched abundant transcript	Up	NEAT1 is essential for the integrity of the nuclear paraspeckle substructure.
51A	an antisense site of intron 1 of SORL1 gene	Up	Downregulating SORL1 variant A
LRP1-AS	Transcribe from the antisense protein- coding LRP1 gene	Up	Inhibit LRP1 expression
XIST	X inactive specific transcript	Up	Regulate BACE1 expression
EBF3-AS	Transcribe from the antisense protein- coding EBF3 gene	Up	Reverse the apoptosis induced by Ab25-35
MALAT1	Promote cell proferation and metastasis	Up	Prevent neuron apoptosis
BDNF-AS	Transcribe from the antisense protein- coding BDNF gene	Up	Prevent neuron apoptosis
NAT-Rad18	Transcribed from the antisense of proteincoding gene Rad18 AD	Up	Down the expression of DNA repair protein Rad18 resulting the neuron more sensitive to apoptosis
SNHG1	small nucleolar RNA host gene 1	Up	Act as a sponge targeting zinc finger gene 217 (ZNF217) of Mir-361-3p
RPPH1	ribonuclease P RNA component H1	Up	Regulate the Aβ 25-35-induced apoptosis and ERS attenuation
MEG3	maternally expressed gene 3	Down	Prevent neuron apoptosis
17A	Embedded in the human G-protein- coupled receptor 51 gene AD	Up	Impair GABAB signaling pathway by decreasing GABAB R2 transcription
ATB	Transforming growth factor	Up	Regulate miR-200/ZNF217 aixs
SOX21-AS1	As a biomarker of neurodegeneration	Up	Downregulate FZD3/5

#### 2.1.2 LncRNA BC200

BC200 (Table 2) is a kind of lncRNA that is highly expressed in nerve cells and various tumors and can significantly inhibit the expression of BACE1; moreover, it has been reported to be abnormally expressed in AD [42] (Figure ). Recent studies have found that an abundance of BC200 is not greatly correlated with sex, age, and years of education, but with the course of AD; further, its expression level in AD is considerably higher than that of the controls. Li et al. [43] found that BACE1 showed down-regulated expression after BC200 siRNA transfection, and reducing the expression of BC200 by siRNA might be a valid approach to prevent AD.

#### 2.1.3 LncRNA NEAT1

LncRNA NEAT1 (Table 2) is a transcriptional product of the *MEN1* gene. It plays a crucial role in the development and maintenance of mammalian cells and can encourage cancer development as well as promote the growth and metastasis of tumor tissues [44]. In recent years, NEAT1 was also found to play a crucial role in central neurodegenerative diseases. Mir-124, which has been shown to act as a regulatory factor to alleviate cell damage in AD by targeting BACE1 expression, is negatively correlated with NEAT1[45] (Figure ). In future, AD can be treated by inhibiting any of the targets in the NEAT1/miR-124/BACE1 axis [46]. In addition, Ke et al. found that NEAT1 aggravates neuronal injury induced by Aβ by regulating Mir-107 and is likely involved in the development of AD [47]. The *CDK5R1* gene encoding P35 has a role in many stages of brain functional development, and its regulation is strongly linked with the occurrence and progression of AD. In 2018, Spreafico et al. observed that the expression abundance of NEAT1 was up-regulated in the temporal cortex and hippocampus of AD patients, and there was a close positive correlation between the abundance of CDK5R1 and NEAT1 in nerve tissues, indicating that NEAT1 may have a neuroprotective effect to compensate for the increased level of CDK5R1 [48]. In 2019, Wang et al. [49] found that NEAT1 could regulate AD by inhibiting and down-regulating the *H3K27AC* gene or up-regulating the *H3K27CRO* gene by inhibiting acetyl-CoA production in Aβ deposition, thereby providing a new intervention target for preventing or delaying the occurrence of AD.

**Figure 2.**
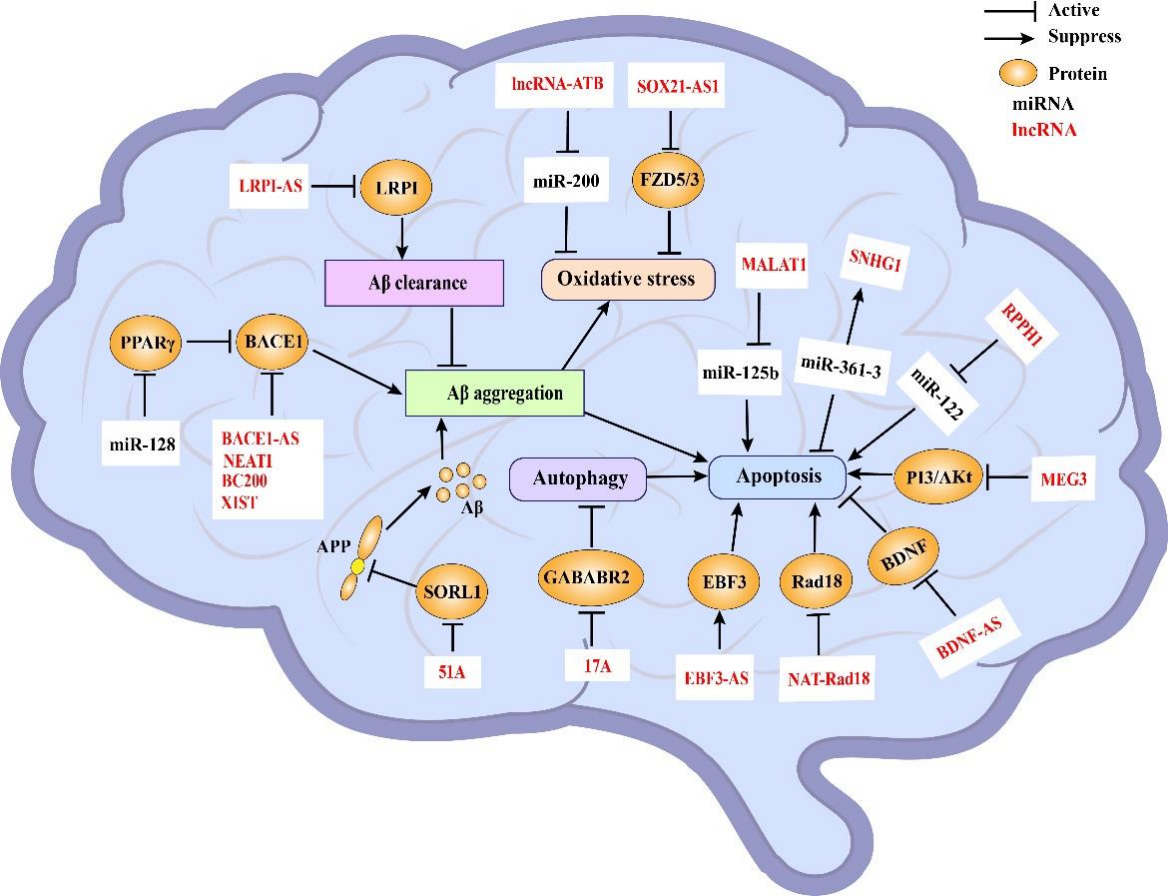
The effects of lncRNAs on pathophysiology in AD.

#### 2.1.4 LncRNA 51A

LncRNA 51A (Table 2), a newly discovered lncRNA, is an antisense site of intron 1 of the sortillin-associated receptor 1 (*SORL1*) gene. *SORL1*, a multifunctional endocytic receptor participating in APP transport [50], is a potential genetic predisposing factor of AD. SOLA encoded by *SORL1* blocks the transport of Aβ APP from Golgi cells to the endosomal compartment containing Aβ secretase [51]. LncRNA 51A regulates the splicing transfer of pre-SORL1 mRNA from the long variant A to the less alternative splicing form (Figure ) [52]. Gómez-Tortosa et al.’s study showed that blocking SORL1 promoted the amyloidosis process and greatly increased the risk for developing AD [53]. 51A was also found to be significantly up-regulated in the human brain, especially in the cerebral cortex of patients with AD [54], suggesting a potential biological target and therapeutic site for AD.

#### 2.1.5 LncRNA LRP1-AS

LRP1-AS (Table 2) inhibits the expression of LRP1, which is a receptor involved in many cellular physiological processes such as intracellular signaling, lipid homeostasis, and APP formation and transport (Figure ) [55, 56] LRP1-AS directly binds to high mobility group box 2 (*HMGB2*) and inhibits its activity, thereby reducing LRP1 mRNA-dependent SREBP1A transcription. Short oligonucleotides targeting LRP1-AS inhibit its interaction with HMGB2 and retain the ability of SREBP1A to drive *LRP1* gene transcription. Interestingly, LRP1-AS is up-regulated in the AD brain; therefore, inhibition of LRP1 could be a neo-treatment for AD.

#### 2.1.6 LncRNA XIST

X inactive specific transcript (*XIST*) is a gene involved in the initiation, transmission, and maintenance of X inactivation (Table 2) [57]. Many studies [58, 59] have found that XIST reduces nerve cell damage in neurodegenerative diseases, and silencing XIST [60] promotes cell degeneration.Yue et al. [61] found that in an AD model(Bilateral common carotid artery occlusion (2VO)-induced-AD model), the expression level of lncRNA XIST was significantly up-regulated *in vivo* and *in vitro*. In N2a cells, silencing of lncRNA XIST negatively regulates Mir-124 and positively regulates BACE1 expression, promoting the progression of AD (Figure ). LncRNA XIST may be a new potential target for the treatment of AD.

### 2.2 Control neuronal apoptosis

#### 2.2.1 LncRNA EBF3-AS

The expression of lncRNA EBF3-AS (Table 2) was reportedly abundant in the AD brain compared to the controls[62]. Further, the expression level of EBF3-AS in the hippocampus of APP/PS1 transgenic rats was higher than that of the normal rats. This indicates that EBF3-AS may play a crucial part in AD, and the abundance of EBF3-AS lncRNA maybe have an effect on the successful establishment of an AD model. Gu et al. evaluated the functions of lncRNA EBF3-AS in Ab25-35 as well as okadaic acid (OA) causing apoptosis of SH-SY5Y neuroblastoma cells [63]. They found that EBF3-AS knockdown significantly reversed the apoptosis induced by Ab25-35 in SH-SY5Y cells (Figure ). This approach may also be used for AD intervention and treatment in the future.

#### 2.2.2 LncRNA MALAT1

LncRNA MALAT1 (Table 2), located on chromosome 11q13, is a long intergenic non-coding RNA (lincRNA) comprising 8828 nucleotides [62, 64]. In recent years, lncMALAT1 has been extensively studied in various tumors, wherein it has been shown that lncMALAT1 can promote cancer cells’ growth and metastasis [65]. In addition, a number of studies have reported that lncMALAT1 has protective effects on nerve ischemia-induced hypoxia injury [66]. Furthermore, its anti-inflammatory effects are observed in other neurodegenerative diseases such as Parkinson's disease (PD) [67]. Ma et al. found that lnc-MALAT1 could influence miR-125b to prevent neuronal apoptosis as well as promote neurogenesis in AD (Figure ) [68].

#### 2.2.3 LncRNA BDNF-AS

Brain-derived neurotrophic factor (BDNF) is a neurotrophic factor expressed in the central nervous system [69]. Considering its critical function in maintaining neuronal activity and synaptic plasticity, BDNF is closely involved in the pathogenesis of AD[70]. Studies have shown that BDNF is down-regulated early in the pathogenesis of AD, which is related to the deposition of Aβ. The toxic effect of Aβ on BDNF expression may lead to synaptic loss and neurodegeneration [71-73]. Moreover, the degree of BDNF down-regulation is related to the severity of cognitive dysfunction. These results suggest that the decrease of BDNF may be the underlying cause of neuronal dysfunction in AD. BDNF pretreatment has potential protective effects on AD [74]. BDNF-AS (Table 2) participates in the progression of AD by inhibiting BDNF (Fig. 2). In addition, inhibition of BDNF-AS increases mRNA expression levels encoding *GDNF* and *EPHB2*, both of which are associated with the pathogenesis of AD [75]. Guo et al. [76] found that blocking BDNF-AS has protective effects to enhance survival viability, reduce apoptosis and prevent oxidative stress of Aβ25-35-induced cell lines under BDNF negative control(Figure ).

#### 2.2.4 LncRNA NAT-Rad18

Rad18 is one of the upstream groups of Rad6, responsible for DNA repair after replication (Table 2). The *NAT-Rad18* gene encodes a natural antisense transcription of Rad18, encodes a series of DNA damage factors, and participates in the regulation of cell apoptosis. Apoptosis is the main form of programmed cell death, disturbance of which can lead to many neurodegenerative diseases including AD. Studies [77] have shown that after Aβ exposure, *NAT-Rad18* is differentially up-regulated to varying degrees in the nervous system, especially in the cortex (Figure ). Taken together, these results suggest that *NAT-Rad18* might participate in the development of AD through its functions on DNA repair systems. This provides new ideas for identifying therapeutic targets of AD [78].

#### 2.2.5 LncRNA 17A

LncRNA 17A is a 159-nt length lncRNA synthesized by RNA polymerase III, located at intron 3 of the *GPR51*gene (Table 2). It can increase the number of GABA-B2 receptor subtypes by alternative splicing [79]. GABABR plays a key part in regulating microglia function as well as neuroinflammation, and GABAB2 is involved in inhibiting synaptic transmission. Another study [80] found that lncRNA 17A (Figure ) is upregulated in AD patients, which weakens GABA B signaling pathway, enhances Aβ secretion, and increases the Aβ X-42/Aβ X-40 ratio. These results indicate that it could directly or indirectly participate in the development of AD and be used to treat AD in the future.

#### 2.2.6 LncRNA SNHG1

Small nucleolar RNA host gene 1 [81] (*SNHG1*,Table 2) is a widely and highly expressed lncRNA in a variety of diseases, and is considered a gene that regulates tumor progression, such as in lung cancer [82]. A number of studies [83-86] have reported that silenced SNHG1 can inhibit neuronal death in a variety of neurodegenerative diseases. In addition, Gao et al. [87] reported that *SNHG1* can act as a sponge targeting zinc finger gene 217 (*ZNF217*) of miR-361-3p, while miR-361-3p itself can inhibit the accumulation of Aβ (Figure ) The overexpression of miR-361-3p reverses the promoting effect of *SNHG1* overexpression on cell damage, and the silencing of *ZNF217* also reverses the promoting effect of miR-361-3p inhibitor on cell damage. Amplifying the role of *SNHG1* as a sponge for miR-361-3p may reduce the cognitive impairment of patients. The role of SNHG1 in AD may provide new ideas for alleviating the progression of AD.

#### 2.2.7 LncRNA RPPH1

Ribonuclease P RNA component H1 (RPPH1, (Table 2), as one of the lncRNAs, is an RNA subunit of RNase P, which is involved in tRNA maturation and sometimes serves as an internal reference gene [88]. Some researchers [89] have found that the *RPPH1* gene is highly expressed in the brain and may be involved in the early compensatory phase of AD [90]. Further, Gu et al. [91]found both lncRNA RPPH1 and miR-122 were up-regulated in AD mice. In Aβ-induced SK-N-SH cells, overexpression of *RPPH1* or inhibition of miR-122 could restore cell viability or reduce apoptosis rate. Dual luciferase reporter gene assays showed that mir-122 directly targets RPPH1 and Wnt1’s 3'-UTR, activates the Wnt/β-catenin signaling pathway, and ameliorates amyloid-β-induced neuronal apoptosis in SK-N-SH cells. In addition, another study [92] found that RPPH1 can directly target mir-326, thereby counteracting its inhibitory effect on PKM2 expression and down-regulating ER stress-related proteins such as GRP78, CHOP, and cleaved caspase-12, contributing to Aβ 25-35-induced apoptosis and ERS attenuation (Figure ). Thus, lncRNA RPPH1 may be involved in the progression of AD through the above mechanisms and may be used as therapeutic targets in the future.

#### 2.2.8 LncRNA MEG3

Maternally expressed gene 3 (*MEG3*, (Table 2) [93], a human homolog of the mouse maternal imprinted gene has been found to be related to the occurrence and progression of a variety of tumors [94], and is also highly expressed in nervous tissues [95], which may be involved in the process of a variety of neuro-related diseases including AD. Yi et al. [96] (Figure ) found that lncRNA MEG3 is down-regulated in AD rat tissues, and up-regulated MEG3 can inhibit oxidative stress injury and inflammatory injury of AD rats, inhibit pathological injury and apoptosis of hippocampal neurons, and reduce the positive expression of Aβ via inactivation of the PI3/Akt pathway, thereby improving spatial learning and memory ability of mice.

### 2.3 Implication in oxidative stress

#### 2.3.1 LncRNA ATB

LncRNA ATB (Table 2) has been confirmed to participate in a variety of pathological process such as in pancreatic cancer and colorectal cancer. Wang et al. found that lncRNA-ATB is highly abundant in AD patients compared to normal subjects (Figure ). Inhibition of lncRNA ATB may protect PC12 cells from Aβ25-35-induced neurotoxicity by regulating the mir-200/ZNF217 axis, which might provide novel ideas for treatment of AD [97]. Notably, mir-200b/C plays a crucial part in reducing the generation of Aβ and Aβ-induced cognition impairment in AD groups by provoking the insulin signaling pathway [98]. Thus, lncRNA-ATB may be involved in the occurrence of AD through the regulation of mir-200.

#### 2.3.2 LncRNA SOX21-AS1

SOX21-AS1 (Table 2), a recently discovered lncRNA, is characterized by its low expression and has been described as a good prognostic biomarker for cervical, oral, and colorectal cancers [99-101]. Sandberg et al. found that SOX21-AS1 showed abnormal expression in the nervous system [102], indicating that it could play a role in neurodegenerative diseases. Zhang et al. [103] silenced SOX21-AS1 and found that it could upregulate FZD3/5 and subsequently activate the Wnt signaling pathway, decrease neuronal oxidative stress, and prevent neuronal apoptosis in AD mice (Figure ).

In addition to the above lncRNAs, there are several others such as CDKN2B-AS1, DLEU1, DLEU2, and DRAIC that are specifically expressed in the nerve tissues of AD patients, found by biogenic analysis. Their exact mechanism in the course of AD has not yet been discovered, but they are likely to play a significant role and can become potentially new targets for the treatment of AD with further research.

## 3. LncRNAs’ roles in AD

### 3.1 Diagnostic biomarkers

LncRNAs exists in the form of multiple exosomes in circulation that are easy to obtain and remain stable under different storage conditions such as at -20? or -80?, physical and chemical conditions, and body fluids, and can be used as novel biomarkers [104]. The roles of lncRNAs have been validated in many cancers [105]. In clinical specimens, exposed lncRNAs isolated from human body fluids are susceptible to degradation due to external factors, while lncRNAs in exosomes can exist stably under the protection of the lipid bilayer in various biofluids, often found in common association with microvesicles or exosomes that protect them from RNAases. These exosomes are potential biomarkers for many CNS injuries including AD [38]. Moreover, due to the non-invasive characteristics of serology, serum lncRNA BC200 is more suitable for screening a large sample size, and lncRNA BC200 is expected to become a biomarker for the diagnosis and efficient monitoring of AD [43, 106]. Feng et al. [107] found that BACE1 in the plasma of AD patients was more abundant than in the plasma of the control group, suggesting that BACE1 may be a potential biomarker to evaluate AD. Many research groups are devoted to validating the potential AD biomarkers to investigate whether lncRNA expression is related to clinical features and prognosis.

### 3.2 LncRNA-targeted drugs

#### 3.2.1 Small interfering RNAs (siRNAs)

SiRNAs that are complementary to the target lncRNAs can be used to target lncRNAs, which recruit arginine-containing RNA-induced silencing complex (RISC) to promote lncRNA degradation. Currently, siRNA has been successfully used in a variety of preclinical models to investigate the prospects of targeted treatment of lncRNA in various diseases. For example, siRNA targeting lncRNA NEAT1 may have a strong inhibitory effect on colon cancer cells, greatly accelerating their apoptosis [108]. The world’s first siRNA-based drug Patisiran was approved in 2018 for the treatment of patients with poly neuropathy caused by genetically induced parathyroid amyloidosis [109], while another siRNA-based drug Givosiran was approved in 2020 for adults with acute intermittent porphyria [110]. However, siRNA still faces one major challenge: it is off-target [111], which means that the target site cannot be identified owing to genetic mutation of the target drugs. While low doses and modified siRNA can alleviate this problem, off-target effects have not yet been fully resolved [112]. Once this limitation is addressed, siRNA targeting lncRNA therapy will have potential therapeutic benefits.

#### 3.2.2 Antisense oligonucleotide

Antisense oligonucleotides (ASOs) are a class of molecular drugs regulated at the genetic level by sequences specifically binding to target gene DNA or mRNA to inhibit gene expression [113]. They can bind to complementary RNA as well as recruit RNase H, inducing RNA degradation and altering downstream protein expression. In the last decade, ASO-related treatments have led to significant clinical breakthroughs. The FDA has approved several ASO drugs on the market for neurodegenerative diseases [114]. For instance, nusinersen is approved to treat multiple forms of spinal muscular atrophy, indicating that ASOs could be the next important direction in neurological disease therapy [113]. Currently, the application of ASOs mainly focuses on the treatment of neurodegenerative diseases like PD [115] and cancers. For example, in 2019, Wang et al. found that ASOs of HAND2-AS1 against BMPR1A have synergistic anti-tumor functions [116]. Nevertheless, ASO, as a special nucleic acid drug, also has a strong off-target effect. Therefore, although there are many pre-clinical utilizations of targeted lncRNAS, further research is still needed to achieve improved results [113].

#### 3.2.3 Clustered regularly interspaced short palindromic repeats (CRISPR)/CRISPR-associated protein 9 (Cas9) system

The CRISPR/Cas9 system is an adaptive immune defense mechanism developed over a long period of evolution in prokaryotes such as bacteria and archaea. By integrating invading phage and plasmid DNA fragments into CRISPR, the corresponding CRISPR RNA (crRNA) was used to induce the degradation of homologous sequences to resist the invasion of viruses and foreign DNA [117]. The CRISPR/Cas9 system consists of sgRNA (single-guide RNA) and the Cas9 enzyme. It works by binding crRNA (CRISPR-derived RNA) to tracrRNA (trans-activating RNA) through base pairing to form tracrRNA/crRNA complexes. This complex guides the nuclease Cas9 protein to splice double-stranded DNA with the sequence target of crRNA pairing. By artificially designing these two RNAs, sgRNA with guiding function can be transformed into sgRNA, which is sufficient to guide Cas9 in site-specific cleavage of DNA [118]. As an RNA-directed dsDNA-binding protein and nuclease, Cas9, was the first known unifying factor that co-located RNA, DNA, and proteins, thus possessing great transformative potential. Fusion of the protein with nuclease-free Cas9 (Cas9 nuclease-null) and expression of the appropriate sgRNA can target any dsDNA sequence, and the sgRNA ends can be attached to the target DNA without affecting Cas9 binding. Therefore, Cas9 can deliver any fusion protein and RNA to any dsDNA sequence, which has great potential for research and modification of organisms [119]. CRISPR/Cas9 has been successfully used for lncRNA targeting because of its advantages of accuracy, efficiency, persistence, and easy-programming, providing inspiration for lncRNA studies[120, 121]. It has been used to treat cancer. Li et al. found that the knockdown of LINC00341 with CRISPR-CasrX inhibited bladder cancer cell growth, triggered cell apoptosis, and inhibited cell activity [122]. Furthermore, Zhao et al.’s [123] study found that the CRISPRi technique could be used in male oligoasthenospermia caused by varicocele via lncRNA. However, similar to siRNA and ASO, CRISPR/Cas9 has the disadvantage of off-target effects. Therefore, until these off-target effects, address system delivery, and other potentially tricky issues can be suitably addressed, we cannot achieve successful clinical application, especially for treating human diseases. Further studies will be required [124].

### 3.3 Exosome-mediated delivery

Exosomes are membrane-derived vesicles that range in size from 20 to 200 nm and are secreted into body fluids, including blood, urine, sweat, and ascites. The contents of exosomes include DNA, protein fragments, and codes or ncRNAs released by the parental cytoplasm and could be absorbed by recipient cells. Recently, one study showed that exosomes from chemotherapy-sensitive cells are capable of significantly influencing recipient cell chemistry through the transfer of specific genes, including lncRNAs [125]. The potential advantages of exosomes for treatment are as follows: (i) exosomes are produced from their own cells, so they cannot easily trigger an immune response; (ii) Exosomes can pass the blood-brain barrier through receptor-mediated endocytosis, thus improving the bioavailability of exosomes in the brain; (iii) Exosomes are nano-sized, which reduces the possibility of microvascular thrombosis; and (iv) Exosomes can be filtered and sterilized, thus reducing the chance of infection [126].

Exosomal-mediated lncRNA has been used in some cancers especially breast cancer. In 2018, Dong et al. found that lncRNA AFAP1-AS1 developed trastuzumab resistance in breast cancer cells by exosome packaging [127]. In 2020, Han et al. found that lncRNA-SNHG14 could be used as a potential biomarker for breast cancer because exosome-mediated lncRNA-SNHG14 induces trastuzumab resistance in breast cancer cells [28]. Taken together, these factors provide the possibility of exosomes being used for AD treatment[128]. Despite the potential advantages of exosomes in the diagnosis and treatment, there are still many challenges to be overcome for the clinical use of exosomes: (i) the mechanism of precisely inducing cells to secrete exosomes containing specific substances is not clear at present; (ii) how to load bioactive substances into exosomes with maximum efficiency; (iii) how to make exosomes selectively target cells and accurately release their contents, and (iv) the biosafety of exosome therapy also needs to be further clarified. In conclusion, the study of the effects of exosomes in AD will provide a new direction for the diagnosis and therapy of AD[129, 130], but there are still multiple hurdles to overcome.

## Conclusion

In recent years, with the advancements in sequencing technology, several novel lncRNAs involved in the pathophysiology of AD have been identified, and this knowledge provides novel insights to enhance our understanding of the role of lncRNAs in AD [131, 132]. Moreover, some of these lncRNAs have been considered as potential biomarkers and therapeutic targets for AD, although the effect of lncRNAs is complex and relative research on lncRNAs in AD is still limited.

Admittedly, there are still many challenges in this area that need to be overcome. Although a large number of dysregulated AD-associated lncRNAs [133] were identified, the functional characterization of most lncRNAs is still lacking, and it is a huge challenge to identify functionally important lncRNAs from multiple recorded non-coding transcripts. Thus, a comprehensive functional characterization of lncRNAs would be the logical next step. Furthermore, although we are beginning to elucidate the function of a few AD-associated lncRNAs, we have a limited understanding of the principles of lncRNA conservation across vertebrates, the existence of functional domains in the RNA molecule, and the molecular mechanisms that regulate the restricted expression of lncRNAs to cellular compartments, all of which are important limitations for the identification of therapeutic targets with translational potential and the development of targeted strategies. In addition, as a potential target for AD treatment, lncRNA-targeted drugs [62] have been developed such as siRNA, AONs, CRISPR/Cas, and exosomes, among others.

The focus of future research should be on how to prevent off-target effects, address system delivery and other such potentially problematic technical issues, and efficient clinical applications, especially for treating human diseases. In addition, exosome-mediated delivery is also a new approach for the treatment of AD and requires detailed investigation [130]. Importantly, various high-throughput identification methods for novel lncRNAs have been applied to different AD studies, resulting in multiple unrelated lncRNA datasets. Therefore, it is extremely essential to adopt a unified nomenclature of lncRNA genes. Overall, more research is needed to further elucidate the functions of lncRNAs in AD and investigate the full potential of lncRNAs as diagnostic and drug targets.
